# Paediatric Obesity Research in Early Childhood and the Primary Care Setting: The TARGet Kids! Research Network

**DOI:** 10.3390/ijerph9041343

**Published:** 2012-04-16

**Authors:** Julia Morinis, Jonathon Maguire, Marina Khovratovich, Brian W. McCrindle, Patricia C. Parkin, Catherine S. Birken

**Affiliations:** 1 Division of Paediatric Medicine, Department of Paediatrics, The Hospital for Sick Children, University of Toronto, 555 University Avenue, Toronto, ON M5G 1X8, Canada; Email: julia.morinis@sickkids.ca (J.M.); marina.khovratovich@sickkids.ca (M.K.); patricia.parkin@sickkids.ca (P.C.P.); 2 Centre for Research on Inner City Health, St. Michael’s Hospital, 30 Bond Street, Toronto, ON M5B 1W8, Canada; 3 The Applied Health Research Centre of the Li Ka Shing Knowledge, Institute of St. Michael’s Hospital, 30 Bond Street, Toronto, ON M5B 1W8, Canada; Email: jonathon.maguire@utoronto.ca; 4 Division of Cardiology, Department of Pediatrics, The Hospital for Sick Children, University of Toronto, 555 University Avenue, Toronto, ON M5G 1X8, Canada; Email: brian.mccrindle@sickkids.ca

**Keywords:** obesity, pre-school, childhood, primary care, prevention

## Abstract

Primary paediatric health care is the foundation for preventative child health. In light of the recent obesity epidemic, paediatricians find themselves at the frontline of identification and management of childhood obesity. However, it is well recognized that evidence based approaches to obesity prevention and subsequent translation of this evidence into practice are critically needed. This paper explores the role of primary care in obesity prevention and introduces a novel application and development of a primary care research network in Canada—TARGet Kids!—to develop and translate an evidence-base on effective screening and prevention of childhood obesity.

## 1. Introduction

The National Centre for Health Statistics in the United States estimates that 16.9% of children and adolescents age 2–19 years are obese [1]. Similar prevalence rates have been found in Canada, with published rates of overweight and obesity in preschool children at 15%, and 6%, respectively [2,3]. Furthermore, the rate of overweight and obesity in Canadian children has been rapidly increasing in the last 30 years, even among children as young as preschool age [4]. Comorbidities and complications of childhood obesity include type 2 diabetes mellitus, hypertension, sleep apnea, non-alcoholic fatty liver disease, orthopaedic issues such as slipped capital femoral epiphysis and psychosocial concerns such as poor self-esteem and quality of life [5,6,7,8,9,10,11,12,13]. Children who are obese are at high risk of becoming obese adults and have great cardiometabolic risk overall [14,15]. Primary care physicians for children are in a unique position for obesity diagnosis, treatment and prevention [16], however, many report having low confidence in the effectiveness of what they can actually provide for their patients [17]. Evidence based approaches and novel research methodology are critically needed in order to improve obesity prevention through early screening and identification, and prevention interventions for children in primary care practice.

## 2. Primary Care and Obesity Prevention

The primary care setting provides a unique opportunity to identify young children at greatest risk of long-term cardiometabolic disease associated with obesity, who may benefit from early identification and intervention [18]. Providing preventive care during health supervision visits in early childhood is a defining component of pediatric primary care practice [19]. In Canada, the majority of children receive primary health care in community-based primary care physicians’ offices (paediatricians or family physicians). This is especially true for infants and toddlers where, in Ontario, over 85% see a physician for routine vaccination [20]. Thus, physician’s offices would seem to be an ideal venue to collect data from young healthy children and develop interventions for obesity diagnosis, treatment and prevention. As health is the reason parents bring their child to a doctor, primary care physicians are uniquely positioned to introduce parents and children to health research. Furthermore, as a result of their special relationship with their patients and their families, primary care physicians may be highly successful at disseminating knowledge generated from such research. Given that primary care physicians see nearly all pre-school children in Canada, they are in a unique position to screen children who are at risk of later-life cardiovascular disease and implement evidence based prevention interventions—if only this evidence existed. 

## 3. The Importance of Early Childhood in Obesity Prevention

There is compelling reasoning that obesity prevention focus on pre-school aged children as the target age group; the pre-school age provides a unique opportunity to influence an individual’s behaviour as it is a developmental stage where parents still have control over feeding and activity [21,22,23,24]. Positive lifestyle changes may have greater impact when initiated at a young age and this is feasible in the primary care setting [23]. Success in obesity interventions has been shown to be associated with earlier identification and referral for treatment specifically in the pre-school age group. For example, two large scale observational studies of treatment programs for children demonstrated a large magnitude increase in long-term treatment efficacy among obese children who were identified and referred for treatment between 2 and 6 years of age compared with those whose obesity was identified and treated in later childhood [25,26]. Furthermore, studies show that overweight begins at an early age and persists, thus highlighting the need for early intervention [18,27]. There is a growing body of literature on the association of rapid growth in early childhood and heightened risk for the metabolic syndrome and its individual components in adulthood, leading to atherosclerotic cardiovascular disease and type 2 diabetes mellitus [28]. Evidence from longitudinal studies has indicated that body mass index (BMI) in childhood tracks reasonably well from childhood and adolescence (age 6–18) into young adulthood (ages 20–37) [29]. Therefore, it is hypothesized that identifying children at risk at an earlier age may change their obesity trajectory [30,31,32,33].

## 4. Screening for Obesity and Overweight in Primary Care Practice

Disease prevention includes the appropriate use of screening and surveillance tests for early detection of disease. As we address childhood obesity, surveillance and screening at the front line is needed. A recent publication from the US Preventative Services Task Force (USPSTF) recognizes the role of primary care clinicians in surveillance for obesity and overweight especially in children [34]. BMI measurement is considered the primary screening measure for obesity in children, and has been shown to be associated with cardiometabolic risk in both children and adults [14]. The USPSTF recommends screening BMI in children 6 years of age and older. They state that there is a dearth of evidence for use in children younger than six years of age despite the recognition of the importance of early obesity prevention [35,36,37]. Further research is needed to identify the best approach for screening in young children—for example, other measures such as waist circumference have not been characterized in young children, and waist circumference may correlate more specifically to later obesity and cardiometabolic risk over time. Although young children with obesity have higher risk of obesity as adults, most obese adults were not obese children. Screening of early lifestyle determinants of obesity such as eating behaviours, physical activity, and sedentary behaviour over time may be of great importance. There is a need for valid and reliable tools that would enable primary healthcare practitioners to screen for such important determinants of obesity as part of routine health surveillance in young children. 

## 5. Prevention of Childhood Obesity Through Primary Care Intervention

Multiple interventions to prevent early childhood obesity have been conducted in the primary care setting [38,39,40]. Taveras *et al*. sought to examine the effectiveness of intervention practices in primary care through motivational interviewing and educational modules targeting television viewing and dietary intake [41]. After one year, the intervention was effective in reducing television viewing but did not significantly reduce BMI [40,41]. Another randomized controlled trial is currently underway in Stockholm, Sweden, using the primary care setting to target obese parents of newborn infants to change lifestyle factors associated with overweight and obesity; results are not yet available [42]. These studies highlight the novel use of the primary care practice for both innovative research designs as well as education on healthy living. The primary health care system is an underutilized opportunity for prevention interventions. Interventions that engage primary care with other relevant sectors such as schools, public health, and social services may be an important consideration. 

## 6. The Development of a Large Canadian Paediatric Primary Care Based Research Network to Target Childhood Obesity

Much has been written about the advantages of Primary Care Based Research Networks in the United States (PCBRN) [43,44]. The largest paediatric PCBRN is the American Academy of Pediatrics sponsored Pediatric Research in the Office Setting, which has been in existence since 1986 and includes over 1,400 practitioners and 470 practice sites [44]. Such networks have a well-documented history of providing access to healthy, non-referred patient populations as well as the opportunity for large sample sizes and the possibility for follow-up of subjects in longitudinal studies. However, previous large cohort studies of this design have not focused deeply on obesity prevention, or on studies related to determinants of obesity including child and family nutrition, physical activity, sedentary behaviours, or social and environmental determinants of obesity. 

The TARGet Kids! Toronto Area Research Group for Kids is a primary care practice based research network developed to respond to the need for evidence based primary care research in childhood obesity prevention. In Canada, TARGet Kids! is the first network of primary care practices to collect longitudinal data relevant to obesity prevention in healthy preschool children, and to aim to develop the evidence base for obesity prevention interventions through practice based interventions. 

TARGet Kids! has emerged as a PCBRN to address obesity prevention in young children under 6 years of age, in partnership between researchers at the Paediatric Outcomes Research Team, SickKids Research Institute, The Applied Health Research Centre (AHRC), at the Li Ka Shing Knowledge Research Institute, St. Michael’s Hospital, and primary care providers in the Section on Community Paediatrics of the Department of Pediatrics and the Department of Family and Community Medicine at the University of Toronto. 

The development of TARGet Kids! arose out of the recognition of the significant burden of illness of obesity, the potential of early intervention in the pre-school age as an ideal time for obesity surveillance and identification, the recognition of the access to this age group through primary care, and the important role of the primary care practitioner for anticipatory guidance to parents of young children. An improved evidence base for anticipatory guidance specifically targeted obesity related interventions and screening has been highlighted as a key research gap [45]. The potential to improve the growth and health trajectory of a child through primary care, and improve both adult and child health drove the development of TARGet Kids! 

## 7. Methodology & Data Collection

The central data collection unit of TARGet Kids! is the primary care group practice. There are currently five large paediatric group practices in TARGet Kids! and one academic family practice unit. These practices care for a large population of children including routine health maintenance visits, have between 3 to 10 practicing physicians, and include physicians with academic appointments through the University of Toronto. Children are recruited, data collection instruments administered, anthropometric measurements taken and blood obtained all during the child’s routine health maintenance doctor’s appointment by a trained research assistant and phlebotomist embedded into the practice site. 

Development of Survey Tools: The TARGet Kids! instruments aim to measure obesity related factors including child and parent health, family history, dietary intake, physical activity, and sedentary activity. A detailed TARGet Kids! questionnaire regarding subject characteristics (including socio-economic status, ethnicity), parent health, dietary intake, physical and sedentary activity was developed and based on questions used in the Canadian Community Health Survey [46] and is completed by the parent. Child temperament is assessed using the validated questionnaire of child temperament, the Child Behaviour Questionnaire (CBQ) for 3–6 year olds [47]. Data on nutrition is collected using the Nutrition Screening Tool for Every Preschooler (NutriSTEP^TM^). NutriSTEP^TM^ is a parent-administered, 17-item nutrition screening tool that has been developed to assess nutritional risk [48]. The NutriSTEP^TM^ has been validated in multicultural Canadian preschool aged children with a detailed assessment by a Registered Dietitian including nutritional history and a 3 day dietary recall. The Parenting Stress Index is a measure that is designed to identify potentially dysfunctional parent-child systems and includes three scales: parental distress, difficult child characteristics, and dysfunctional parent-child interaction. It is administered at each visit from the age of 3 months to 10 years [49].

Health Measures: Measurement of height, weight and waist circumference of subjects and their mothers are performed using standardized anthropometric protocols [50].

Non-fasting blood samples related to obesity and cardiometabolic risk are drawn by trained pediatric phlebotomists on site, and include the following measures: lipid profile, insulin, glucose, ApoA1, ApoB, CRP, 25-HydroxyVitamin D and ALT, Adiponectin and Leptin [51]. Topical anesthetic cream (EMLA or Ametop) is offered to minimize discomfort from venipuncture. 

Serum samples are sent daily to the Mount Sinai Services (MSS) Laboratory, a centralized research laboratory in Toronto. The MSS laboratory has defined itself through the use of automated sample processing and electronic data handling as the research laboratory for the University of Toronto clinical research. 

Data Management: The TARGet Kids! central database is housed at the Applied Health Research Centre of the Li Ka Shing Knowledge Institute, St. Michael’s Hospital, Toronto. Data entry is accomplished by research assistants at each practice site and uploaded to the central database in real time through a web‐based remote data entry system using the MediData Rave™ platform (MediData Solutions, New York, NY, USA). This ensures reliable, efficient, and secure data handling. Research ethics was approved through the Research Ethics Boards of the two institutions of the lead investigators of TARGet Kids; the Hospital for Sick Children research ethics board, and St Michael’s Hospital research ethics board. The primary care practitioners have appointments through the Faculty of Medicine, University of Toronto, and use these research ethics boards. As TARGet Kids! expand to different jurisdictions, application through relevant research ethics boards will be required.

## 8. TARGet Kids! as a Platform for Child Health Research in Obesity

TARGet Kids! is a surveillance platform: The first child was enrolled into TARGet Kids! in September 2008. Since then as of August 2011, TARGet Kids! has recruited over 3,500 children from 0–5 years of age. Blood sample collection was instituted in December 2008, and laboratory data have been collected and analyzed on over 2,405 children. This is the largest cohort in this age group in Canada recruited from primary care. The mean age of participants at baseline is 33.5 months with the distribution of males at 51.6%. The current TARGet Kids! cohort consists of 1,331 children aged 12 to <24 months (37%), 512 children aged 24 to <36 months (14%), 577 children aged 36 to <48 months (16%), 670 children aged 48 to <60 months (19%), and 441 children aged 60 to <72 months (12%). TARGet Kids! has recently began to recruit participants <12 months of age. 

Prevalence rates among TARGet Kids! participants for common paediatric conditions are consistent with available prevalence data providing evidence for the representativeness of the sample. For example, the proportion of children included in TARGet Kids! with zBMI > 1 is 20.5% and zBMI > 2 is 4.8%, which is consistent with Canadian national data [4].

TARGet Kids! is a platform for cross-sectional studies: A recent study in the TARGet Kids! platform identified factors associated with increased daily screen time in three year old children. Controlling for maternal education and age, eating lunch and dinner in front of the screen and mother being employed were associated with an increase in child weekday screen time of 96, 42, and 36 min/d, respectively. Family rules decreased child weekend screen time by 30 min/d [52]. Other cross sectional studies include examining other sedentary behaviours such as stroller use, and prolonged bottle feeding [53].

TARGet Kids! is a platform for a longitudinal cohort studies: The longitudinal effects of child and parent nutrition, physical activity, and sedentary behaviours, their influence on growth, and cardiometabolic risk can all be determined. Children recruited through TARGet Kids! will be followed on an annual basis at each of their regularly scheduled well child visits. Studies underway include a longitudinal study on vitamin D levels and obesity outcomes, and early growth and cardiometabolic outcomes in early childhood. This efficient approach to follow up capitalizes on scheduled clinical follow up. 

TARGet Kids! is a platform for randomized controlled trials: A pragmatic randomized controlled study in the TARGet Kids! platform was performed at the 9 month visit and was effective in promoting early cup use, bottle weaning, and reduced prolonged bottle feeding [53]. Another TARGet Kids! example includes a pragmatic single blinded randomized controlled trial on screen time. At the 3 year old annual health visit, the treatment group received an educational session on reducing screen time; the control group received a session on safe media use. Follow up was completed at 1 year (4 year old annual health visit) (manuscript submitted for publication) [54]. These trials highlight the use of the primary care setting for trials that enable development of an evidence based for obesity prevention.

Other Data Collection Methods in PCRNS: Electronic Health Records: Other opportunities to generate evidence in primary care may be through the use of electronic health records data for research. The integration of electronic medical records (EMRs) into clinical care is emerging in Canada, but is slow and inconsistent. Indeed there is a variety of EMRs being used in TARGet Kids! current practice sites. We continue to work towards advancing our research through use of EMRs and prepare the groundwork for TARGet Kids! to be poised to utilize this technology effectively. 

## 9. Lessons Learned from the Development of TARGet Kids!

The development of TARGet Kids! as a PCBRN has been an example of an innovative and novel research platform within the field of child health. The challenges that have been faced provide important knowledge for others engaging in primary care research as follows: 

Financial Support: Stable funding for the development of the TARGet Kids! infrastructure is a potential challenge, however, the relevance and rigor has been identified by external reviewers and we have been very successful in receiving funding from various sources including national federal granting agencies for the team grant and operating funds for individual projects (CIHR), other granting agencies (Physician Services Incorporated Foundation, the Danone Institute, Dairy Farmers of Canada), hospital foundations and through private donations. Innovative grants for the development of infrastructure are needed in order to further the development of important research in this area. The development of private-public partnerships may be important moving forward.

Data management: Managing a large set of data could be daunting, however, the TARGet Kids! research has been highly supported by an academic research organization, the Applied Health Research Center (AHRC) which provides a rigorous data management platform of personnel (coordinators, biostatisticians) and an electronic platform (Rave) which has enabled the collection of large quantities of data in order to build the TARGet Kids! database. Furthermore, laboratory information has been managed through Mount Sinai Services Inc. (MSS) which is a global provider of customized laboratory and research services. The MSS allows real-time feedback to the front line health providers as well as appropriate storage and data linking to the TARGet Kids! database.

Communication and team building: Effective communication with all members of the team is key and early collaboration with primary care providers who are also local opinion leaders in the field of pediatric obesity was essential in order to build the TARGet Kids! research platform. The development of a TARGet Kids! steering committee, and regular meetings with all stakeholders is crucial to provide feedback and to allow the front-line clinicians to take a keen role in the development of research based on their own clinical queries. The use of the research assistant within each office practice links the child to the research team however, further ways to build communicative links with health care providers to change health care provider behavior based on novel evidence generated through TARGet Kids! is an area that is being looked at further as a new area of research within the TARGet Kids! platform. 

Academic Recognition: Appropriate academic recognitions for the primary care practitioners has been facilitated through the section of Community Pediatrics within the department of Paediatrics at University of Toronto. Other methods of recognition have not been explored. 

## 10. Opportunities for Knowledge Translation in Primary Care Practice

The TARGet Kids! network provides a novel opportunity for *truly integrated knowledge translation* with primary care physician practice community. The primary care physician members of TARGet Kids! have all participated in the early stages of shaping the research process. Members of the TARGet Kids! network include decision makers and policy makers in primary care practice. Findings from this research are disseminated directly to the physician participants and to their patients during an annual meeting of all the TARGet Kids! practice staff, research team, and policy leaders, and parents of participants will receive summary of validated measure of nutritional risk [48], as well as feedback from their physicians regarding laboratory measures, leading to a direct benefit for individual participants. Further dissemination of knowledge will occur within the academic community through publication in relevant journals and presentations at national and international conferences, and locally through hospital rounds and presentations. 

## 11. Conclusions

There are opportunities to develop and translate obesity prevention knowledge in primary care practice through the development of a research platform in primary care. The TARGet Kids! Research network represents the development of a novel platform for research to further our understanding of obesity prevention through surveillance, cross sectional, longitudinal, and pragmatic randomized controlled trials in order to develop and translate an evidence-base on effective screening and prevention of childhood obesity to improve the health of Canadian children.
